# Understanding age variations in the migrant mortality advantage: An international comparative perspective

**DOI:** 10.1371/journal.pone.0199669

**Published:** 2018-06-29

**Authors:** Michel Guillot, Myriam Khlat, Irma Elo, Matthieu Solignac, Matthew Wallace

**Affiliations:** 1 Population Studies Center, University of Pennsylvania, Philadelphia, Pennsylvania, United States of America; 2 French Institute for Demographic Studies (INED), Paris, France; 3 Univ. Bordeaux, CNRS, Comptrasec, UMR 5114, Pessac, France; University of Miami, UNITED STATES

## Abstract

This paper investigates age variations in foreign-born vs. native-born mortality ratios in an international comparative perspective, with the purpose of gaining insight into the mechanisms underlying the so-called migrant mortality advantage. We examine the four main explanations that have been proposed in the literature for the migrant mortality advantage (i.e., in-migration selection effects, out-migration selection effects, cultural effects, and data artifacts), and formulate expectations as to whether they should generate an increase, a decrease, or no change in relative mortality over the life course. Using data from France, the US and the UK for periods around 2010, we then examine typical age patterns of foreign-born vs. native-born mortality ratios in light of this theoretical framework. We find that these mortality ratios vary greatly by age, with important similarities across migrant groups and host countries. The most systematic age pattern we find is a U-shape pattern: at the aggregate level, migrants often experience excess mortality at young ages, then exhibit a large advantage at adult ages (with the largest advantage around age 45), and finally experience mortality convergence with natives at older ages. The explanation most consistent with this pattern is the “in-migration selection effects” explanation. By contrast, the “out-migration selection effects” explanation is poorly supported by the observed patterns. Our age disaggregation also shows that migrants at mid-adult ages experience mortality advantages that are often far greater than typically documented in this literature. Overall, these results reinforce the notion that migrants are a highly-selected population exhibiting mortality patterns that poorly reflect their living conditions in host countries.

## Introduction

In the context of growing international mobility, health and mortality patterns among migrants are playing an increasingly-important role in many receiving countries, with implications for health care, health insurance schemes, and pension systems [1,2]. Increases in the proportions of foreign-born individuals in receiving countries also imply that mortality patterns among migrants carry an increasing weight on national mortality levels of host countries, potentially affecting international mortality rankings.

In the literature on mortality among migrants, the most pervasive finding is that migrants tend to exhibit lower mortality than the non-migrant population of their host countries. This phenomenon, termed the Migrant Mortality Advantage (MMA), has been observed in a wide variety of receiving countries, including Australia [3,4], Belgium [5,6], Canada [7,8], France [9,10], Germany [11,12], the Netherlands [13], Switzerland [14], the United Kingdom [15–17], and the United States [18–23]. The MMA has been explained using various hypotheses, including in-migration selection effects (“healthy migrant effect”), out-migration selection effects (“salmon bias”), cultural effects, and data artifacts. However the relative contribution of each of these hypotheses in various contexts remains highly debated in the literature.

One limitation of this literature is that it often ignores age variations in the relative risk of mortality among migrants. Mortality or hazard ratios for foreign-born vs. native-born individuals are typically documented over wide or open-ended age groups [17,24–27]. This lack of age detail is perhaps due in part to the increasing reliance on survey data, which rarely have the sample sizes necessary to estimate mortality without relying on parametric or semiparametric assumptions. One such assumption (for example in certain proportional hazard models) is that relative mortality risks between foreign-born and native-born individuals are constant over age. In essence, this ignores possible age variations in mortality ratios. Poisson regression models that simply control for age without interactions, or comparisons of life expectancies, also hide age variations in mortality ratios.

Due to this lack of age detail, conclusions about the existence and scale of the MMA are often made without reference to age. This can give a distorted impression that relative to the native-born population, migrants exhibit a relative mortality risk that remains constant over age. Likewise, explanations of the MMA are often discussed with little reference to age. For example, when discussing the role of migrant selection at entry, the fact that the strength of the selection may vary markedly by age [28,29] is often ignored. However, if it is the main explanation for the MMA and its effects on mortality taper with duration of stay in the host country, we would expect the MMA to be larger at the ages where the proportions of recent migrants are higher [20].

Thus rather than testing hypotheses for the MMA in reference to overall levels of relative mortality, it is more suitable to test such hypotheses in reference to age variations in these relative risks. A few studies in the literature have followed this approach [20,23,30–32], but these studies typically focus on one host country or one migrant group, and they rarely include all ages.

Our aim is to give age patterns more salience by considering the entire age range and relating age variations in the MMA to the mechanisms that seek to explain it. In doing this, we will advance our understanding of the MMA and the underlying mechanisms which generate it. In addition, our approach is considerably enriched with an international comparative perspective, something which is rarely done in this literature. Indeed, if a general set of mechanisms truly do work to influence the advantage, we should observe some consistency in the shape and scale of the MMA for different migrant populations across a variety of contexts.

This paper is organized as follows. First, we examine the four main hypotheses that have been proposed in the literature to explain the MMA: (1) in-migrant selection effects; (2) out-migrant selection effects; (3) cultural effects; and (4) data artifacts. In turn, we discuss whether each explanation would be expected to generate an increase, decrease, or no change in the MMA over the life course.

Second, we examine typical age variations of foreign-born vs. native born mortality ratios, using data from three major host countries: France, the UK and the US. Our methodological approach relies on unlinked death information (from death registration) and exposure information (from censuses), by sex and country of birth, for five-year age groups from ages 5–9 until ages 85+, for periods around 2010. We focus on unlinked census and death registration data, rather than on linked data sets, because their large sizes allow us to detect age variation of relative migrant mortality with more precision. We calculate mortality ratios by country of origin to examine the extent to which age patterns follow regularities or are highly specific to each country of origin.

Finally, typical age patterns of migrant relative mortality found in France, the UK and the US are examined in light of our theoretical framework. We discuss how consistent each explanation is with the observed age patterns. We pay particular attention to explanations that are consistent with overall, age-adjusted risk ratios but do not hold once age variations in risk ratios are taken into account.

## Theoretical framework

In this section, we examine the different hypotheses that have been proposed to explain the MMA, and discuss how they might be expected to operate over age. We focus specifically on how these hypotheses impact mortality outcomes at the aggregate level, i.e., how they produce variation over age in observed ratios of foreign-born vs. native-born mortality rates. This means that we need to address both: (1) factors operating at the individual level, such as the effect of migrant selectivity and duration of residence in the host country on the individual risk of death; and (2) composition effects, resulting from changes with age in the composition of migrants with respect to factors such as selectivity and duration of residence. These compositional changes arise in part from the dynamics of entries and exits in and out of the migrant population over age. Our theoretical framework addresses both phenomena.

### In-migration selection effects

In-migration selection effects (also sometimes referred to as the “healthy migrant effect”) is one of the major explanations for the MMA [7,11,24,30,33–37]. According to this explanation, individuals who migrate may be more robust, on average, than members of the sending population, and this selection may be strong enough such that migrants end up being also more robust, on average, than members of the receiving population, generating a mortality advantage.

When examining how in-migration selection effects may impact age variations in the relative mortality of migrants, two individual-level processes need to be considered. First, positive health selection is likely to be most relevant for individuals who migrate for study or work, for whom individual characteristics play a more important role in the migration process, and less relevant for individuals who migrate through family reunification, for whom individual characteristics play a less important role [38]. This implies that positive health selection will be most relevant for migrants arriving at young adult ages, and less relevant for migrants arriving as children or as older adults near or at retirement ages. Second, any effect of in-migration selection on mortality is likely to be most important shortly after migrating and less important as duration of stay in the host country increases. Indeed, in-migration selection at younger ages does not imply protection throughout the life course, and thus a migrant’s level of robustness on the day of his/her arrival to the receiving country may not be so relevant for predicting his/her mortality in the receiving country 20 or 30 years later [20,36].

**Fig 1:**
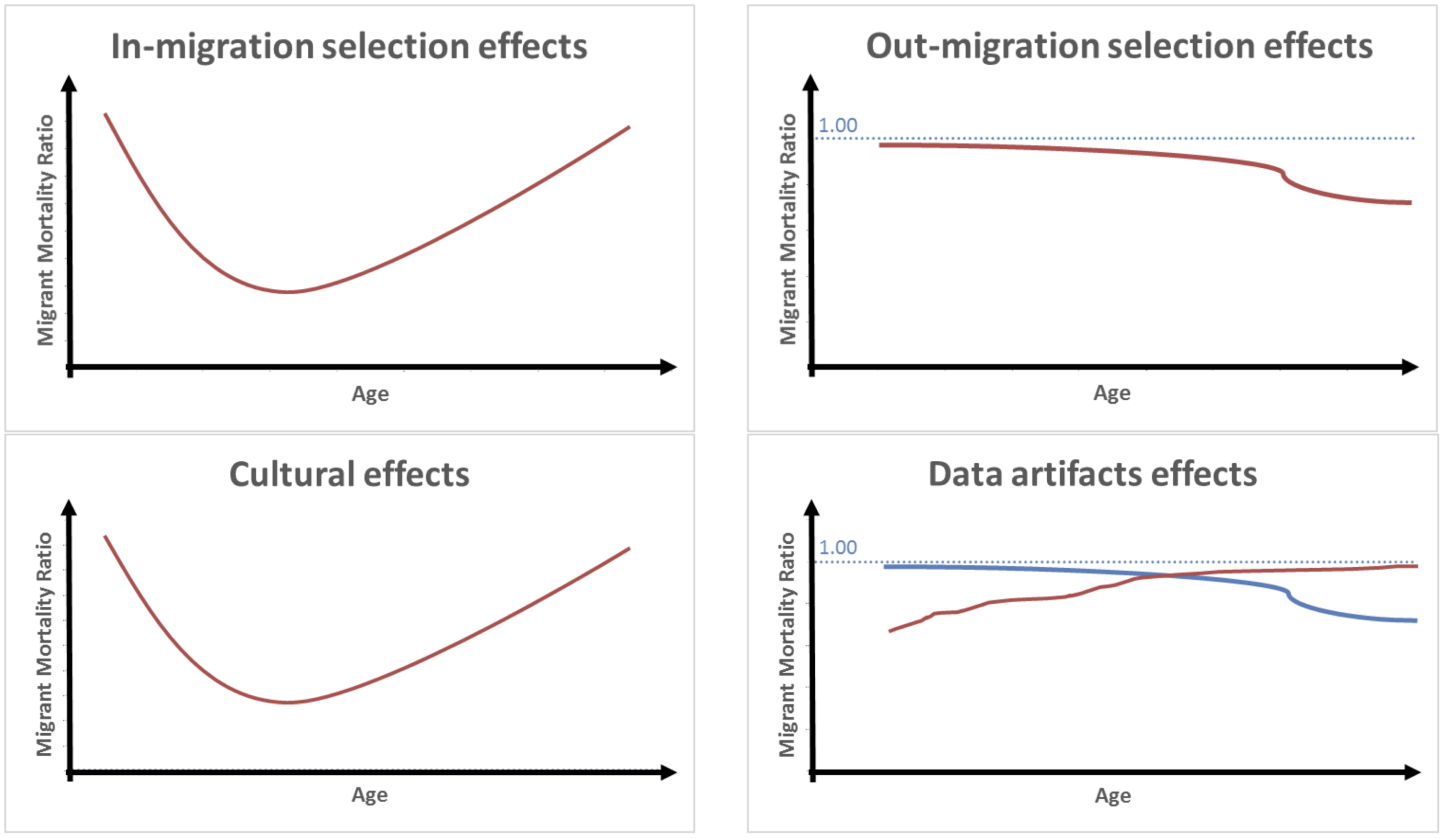
Expected independent effects of major explanations for the migrant mortality advantage on age variations in mortality ratios (Foreign-born vs Native-Born).

The combined effect of these two processes on the MMA can be hypothesized to operate as follows. First, the size of the advantage should be smaller at younger ages (say, below 18) where foreign-born individuals are less subject to positive health selection. Second, the advantage should initially strengthen with age, as large numbers of more positively-selected individuals arrive in the host country for study or work. Finally, the advantage should diminish with age as fewer migrants arrive (often to join their children) and the average duration of residence of current migrants increases. Another factor which will contribute to the erosion with age of the effects of in-migration selection on the MMA is mortality selection. As age increases, less robust individuals among both migrant and non-migrant sub-groups in the host country will be gradually weeded out, making the frailty composition of these two sub-groups more similar and thus producing mortality convergence [30,39]. This convergence with age in the mortality of migrants vs. non-migrants arising from mortality selection is expected to happen regardless of adaption/assimilation processes [39]. Overall, when considering in-migration selection effects alone, we expect the relative mortality of migrants to follow a U-shape pattern over age. This is illustrated in Fig 1 (upper-left panel).

### Out-migration selection effects

The “out-migration selection” explanation, also referred to as the “salmon bias” hypothesis, postulates that migrants in poor health in the host country may be more likely to return to their country of origin than migrants in good health, for reasons ranging from the willingness to seek better family support to the desire to die in one’s birthplace [18,20,30,36,37,40,41]. As a result of this “unhealthy remigration,” foreign-born individuals remaining in the host country may have better health, on average, than would be observed in the absence of out-migration, potentially contributing to the MMA.

Out-migration selection effects (net of other factors) can be expected to impact age patterns of the MMA as follows. Overall, the gradual removal over age of individuals in poor health from the risk pool is expected to generate a continuous decrease with age in the relative mortality of migrants. However, if unhealthy remigration is indeed taking place, we expect the declines to be larger at older ages, when increases in morbidity generate a larger pool of migrants potentially subject to this phenomenon [20]. This is illustrated in the top-right panel of Fig 1.

### Cultural effects

The “cultural effects” explanation for the MMA posits that migrants may practice more favorable health behaviors (e.g., smoking, alcohol consumption, and diet) than the non-migrant population due to the prevailing cultural norms in their country of origin [36,42–44]. These more favorable health behaviors may generate lower mortality among migrants [25,30,45,46].

Additionally, the cultural effects explanation suggests that migrants may have better health outcomes than non-migrants because they tend to benefit from denser social support networks, including stronger family ties [20,28,47,48]. These denser social networks produce “cultural buffering,” which may be particularly protective against the risk of coronary heart diseases [28,49,50].

In order to hypothesize about how cultural effects may operate by age, the following mechanisms can be raised. First, cultural factors should have an effect on the MMA starting primarily at ages where health behaviors such as smoking and alcohol consumption, or causes such as coronary heart diseases, are relevant for explaining mortality outcomes. This will largely exclude younger ages, say, below 20. Second, we expect cultural effects to be most relevant among recent migrants who are most likely to adhere to the norms and behaviors of their country of origin and benefit from strong immigrant social support networks. Third, given the expectation that migrants will experience some degree of acculturation, cultural effects should attenuate over time as duration of residence in the host country increases. Indeed, with increasing duration of residence, it is expected that the health behaviors of migrants will become more similar to those of the host population, and the degree of protection provided by immigrant social networks will attenuate [51–56].

In combination, these different processes can be expected to produce a U-shape pattern on the relative mortality of migrants, similar to in-migration effects. Cultural effects should first generate a decrease with age in the relative mortality of migrants, as health behaviors become increasingly relevant for mortality and the proportion of recent migrants increases following a peak in arrivals in the early 20’s. Cultural effects should then generate an increase with age in the relative mortality of migrants, as new arrivals decrease and the mean duration of residence in the host country increases. This is illustrated in the bottom-left panel of Fig 1. Because age patterns arising from cultural effects are similar to age patterns arising from in-migration selection effects, it will be difficult to differentiate between the two mechanisms when examining empirical patterns. We will address this issue in more detail when interpreting our results in the Discussion section below.

### Data artifacts

Data artifacts are often raised as an explanation for the MMA [5,20,30,36,37]. Indeed, the estimation of mortality among the foreign-born population is subject to a number of data problems that are inherent to the very nature of the migrant population: a population that is highly mobile and difficult to capture correctly in data sources.

Consistently with the data we use in this paper, we focus here on data quality issues that are relevant for mortality estimates based on unlinked deaths and population information, and where the origin of foreign-born individuals is based on country of birth information. Classic data problems in this literature, such as matching bias or censoring bias at the individual level, are not directly relevant when examining unlinked data. Likewise, the numerator/denominator bias, which is a critical problem when using race/ethnicity to determine the origin of migrants [57], is not so relevant when the origin of migrants is determined on the basis of country of birth information, a basic demographic variable that is less subject to response bias.

Consequently our discussion of data artifacts and their impact on the age pattern of the MMA focuses on the following remaining issues: (1) coverage of deaths; (2) coverage of the population; (3) age misreporting in death registers and/or population estimates. We also focus our discussion on how these issues affect mortality estimates specifically for the foreign-born population. While mortality estimates for the native-born population in host countries may not be perfect, it seems reasonable to assume that age-specific variations in the MMA are not primarily explained by data quality issues among the native-born population.

In theory, age-specific mortality rates and resulting life tables are calculated for the resident (“de jure”) population of a country. This means that both deaths and person-years of exposure should pertain to the resident population, regardless of the “de facto” location of these deaths and person-years. In practice, however, there is typically a “de facto/de jure” mismatch between the numerator and the denominator of mortality rates. While person-years of exposure are typically based on “de jure” population estimates, counts of deaths typically follow a “de facto” definition: they include deaths of non-residents occurring within the boundaries of a country, and exclude deaths of residents occurring outside these boundaries [58]. (National vital registration systems are poorly equipped to gather information on residents dying abroad, especially when these residents have a foreign nationality.) While this mismatch may not generate important errors for the native-born population, it is potentially problematic for the foreign-born population, especially in an era where cheap travel and easy communication facilitate the maintenance of transnational ties as well as back-and-forth travel (sometimes called circular migration) between sending and receiving countries [59]. Indeed, transnationality and circular migration implies that foreign-born residents of a host country are more likely to spend some portion of a given year abroad, which mechanically increases the likelihood that their death will occur abroad and be missing from the numerator of mortality rates. (Deaths that occur abroad following a change of official residence do not pose a data quality problem per se since these deaths and corresponding person-years are not supposed to be accounted for in national mortality rates. However they may affect mortality rates via selection effects—see section above on out-migration selection effects.)

Given this “de facto/de jure” mismatch, it follows that the amount of time per year spent abroad among the foreign-born resident population, and how it may vary by age, is a key factor for understanding the impact of data artifacts on the MMA. Detailed quantitative information about how foreign-born residents divide their time between their host country and their country of birth is lacking. For some foreign-born residents, the amount of time per year spent abroad may decrease with age due to weakening ties with the country of origin, as well as declining health which makes back-and-forth travel more difficult. On the other hand, retirement opens up opportunities for spending more time in the country of origin, and thus for some foreign-born residents, older age may coincide with a larger portion of a year’s time spent abroad. Maintaining official residence in the host country (and being counted as such in data sources) while spending a portion—or all—of a year’s time abroad may also be advantageous for some foreign-born retirees, since in certain host countries benefits such as pensions and health care depend in part on maintaining legal residence.

If the dominant age pattern is one in which the portion of a year’s time spent abroad among foreign-born residents diminishes with age, we expect to observe an artifactual increase with age in the relative mortality risk of migrants. If, however, the portion of a year’s time spent abroad increases with age, this would produce a decrease in relative mortality with age.

For similar reasons, the coverage of the foreign-born resident population may also vary with age. Foreign-born residents who spend large amounts of time abroad are more likely to be undercounted, especially if they live alone or travel with their household members. Census coverage will thus tend to increase with age among foreign-born residents who spend decreasing amounts of time abroad as they age, while it will decrease for those who spend increasing amounts of time abroad as they age. Another factor that will produce an increase with age in the coverage of foreign-born residents is documented vs undocumented status. This arises from the fact that the proportion of undocumented migrants (who are typically less well covered in censuses) can be expected to decrease with age.

The net effect of these errors on age patterns of the MMA is difficult to assess without more specific information on the processes discussed above. Nonetheless it is likely that errors in death coverage will be more consequential than errors in population coverage, because as said earlier deaths occurring abroad among foreign-born residents are systematically excluded, while foreign-born residents travelling abroad may still be included in censuses (e.g., if they are reported by other household members, or if their absence does not coincide with the census date). It can thus be hypothesized that foreign-born residents spending an increasing portion of a year’s time abroad as they age will exhibit a decline in their mortality ratio, while those spending a decreasing portion of a year’s time abroad as they age will exhibit an increase in their mortality ratio.

Age misreporting is another factor potentially affecting the MMA and its age pattern. Migrants from less-developed countries often lack proper documentation about their actual date of birth, contributing to age misreporting both on census records and death certificates. Simulations of various patterns of age misreporting errors (age overstatement, age understatement, and symmetric age misreporting) show that such errors tend to produce death rates that are too low, with increased biases at older ages [60]. As a result, if present, we expect age misreporting to generate a decrease with age in the relative mortality risk of migrants, particularly at older ages. This pattern may be particularly pronounced for migrant groups originating from less-developed countries who are more subject to age misreporting.

This section shows that the effects of data artifacts on the MMA are complex and multidirectional. Nonetheless these effects can be summarized as follows. Data artifacts are likely to produce a decrease with age in the MMA among migrants for whom older age coincides with increased portions of a year’s time spent abroad, or among migrants who are more subject to age misreporting (such as those from less-developed countries). Conversely, they are likely to produce an increase with age among groups with decreased portions of a year’s time spent abroad. These two possible effects are illustrated in Fig 1 (bottom-right panel).

## Data and methods

This paper relies on unlinked deaths and exposure information by age, sex and country of birth, in France, the UK and the US. In all three countries, exposure information is based on “de jure” census counts, while death information is based on “de facto” vital registration data. This is the classic data configuration in national-level mortality estimation [58]. Consistently with international practice, migrants are defined as foreign-born individuals, regardless of their nationality at birth.

For France, we combined death information for the period 2005–2009 with January 1 census estimates for 2006–2009. For the UK, we combined death information for the period 2010–12 with census information for 2011. For the US, we combined death information for 2008–2010 with exposure information derived from the American Community Survey (ACS) for the same period. In France and the UK, country-of-birth information was available by single country in both census and death information. In the US, however, country-of-birth information on death certificates was available only for the following countries of birth: Canada, Cuba, Mexico, and all other countries combined.

Combining death and exposure information, we calculated age-specific death rates (_n_M_x_) by country of birth and sex. We then calculated age-specific mortality ratios for each migrant group by dividing the age-specific mortality rate for a given migrant group by the corresponding age-specific mortality rate for natives: MxnCountryofbirthiMxnNatives. Mortality ratios were calculated for each 5-year age group, from 5–9 until 85+. (The age group 0-4 was excluded due to the small number of foreign-born individuals in that age group.) Confidence intervals were calculated using a Poisson model.

We also calculated age-adjusted risk ratios for various migrant groups using Poisson regression models with age controls. Such age-adjusted risk ratios make the implicit assumption that the relative risk is constant over age, similar to the “proportional hazard” assumption of a Cox regression model. Confidence intervals for these age-adjusted risk ratios were derived from the corresponding Poisson model.

## Results

**Fig 2:**
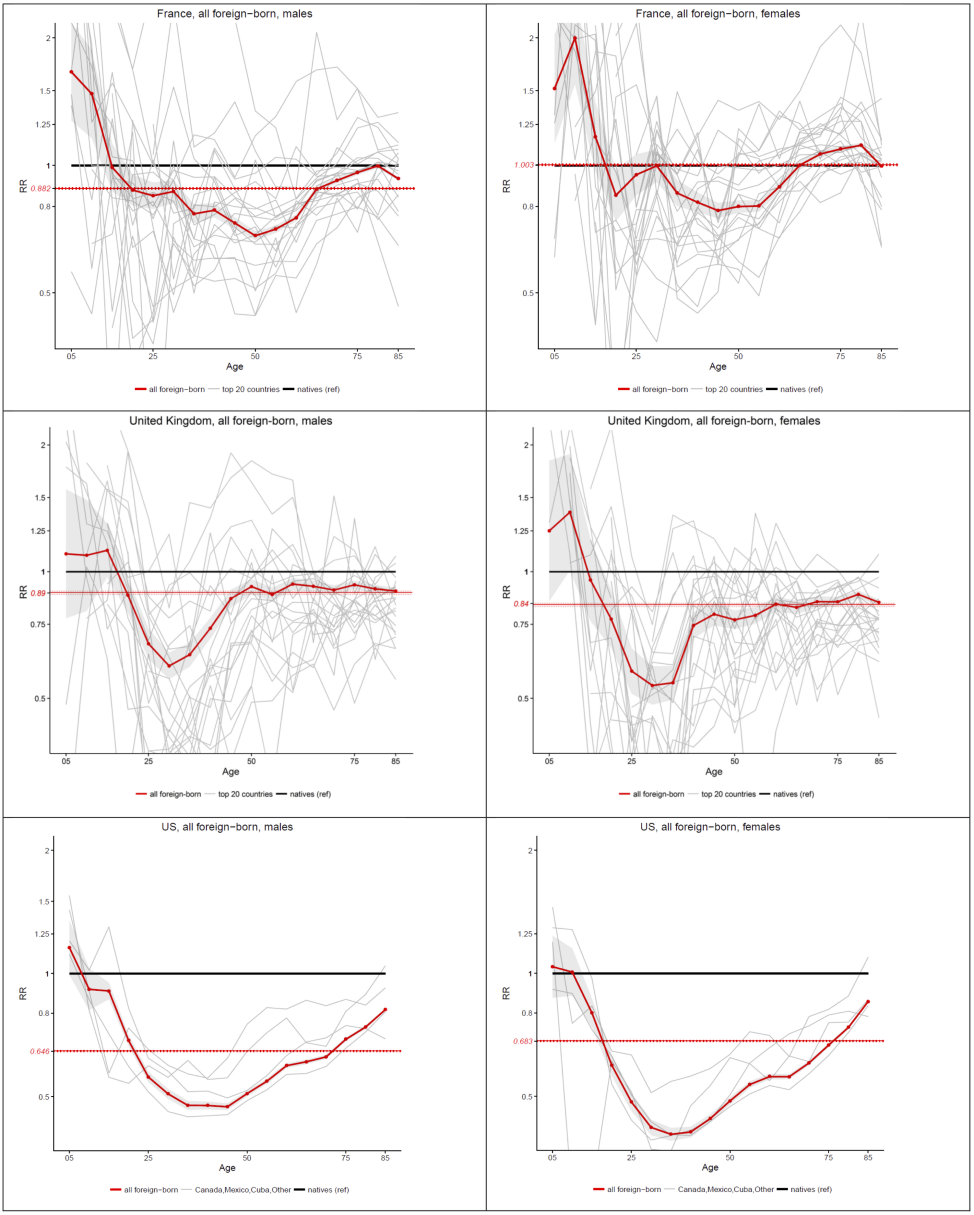
Age-specific and age-standardized mortality ratios (Foreign-born vs Native-Born) in France (2005–09), the UK (2010–11) and the US (2008–10), by sex.

Fig 2 shows age-specific mortality ratios for foreign-born vs. native born individuals in France, the UK and the US, by sex. Ratios above one in this figure correspond to situations of excess mortality for the foreign born, while ratios below one depict situations where the foreign-born exhibit a mortality advantage. The red curve shows age-specific ratios for all foreign-born individuals combined, with 95% confidence intervals, for each host country and sex. The dotted red flat line shows the age-adjusted risk ratio for the foreign-born in the Poisson model, here also with confidence intervals. The gray lines show age-specific risk ratios by individual country of origin (for the 20 most important countries of origin in terms of size of the migrant population in France and the UK).

These results confirm that for almost all country×sex combinations, there is a substantial amount of mortality advantage for the foreign-born, summarized by an age-adjusted risk ratio that is less than one. The only exception is foreign-born females in France, for whom the risk ratio is close to 1. These age-adjusted risk ratios, however, hide a huge amount of age-specific variation, including ages at which there is actually excess mortality, and ages at which the advantage is far greater than what would be indicated by the age-adjusted risk ratio. Fig 2 also shows that in spite of a great amount of variability by country of origin, a systematic U-shape pattern appears in each host country and sex when combining all foreign-born groups. Although not always statistically significant, the risk ratio starts above one at ages 5–9, followed by a steep decline in the ratio until a minimum somewhere around age 45. This minimum value, which varies in each host country, can sometimes be as low as .5, showing an advantage at these mid-adult ages that is far greater than typically documented in this literature. After reaching this minimum, the risk ratio increases towards one, and sometimes even goes above one like in the case of foreign-born females in France. This consistency is striking given the variety of situations among these three host countries in terms of origin of migrants and conditions in the host country. To our knowledge, this consistency has not been previously documented.

**Fig 3:**
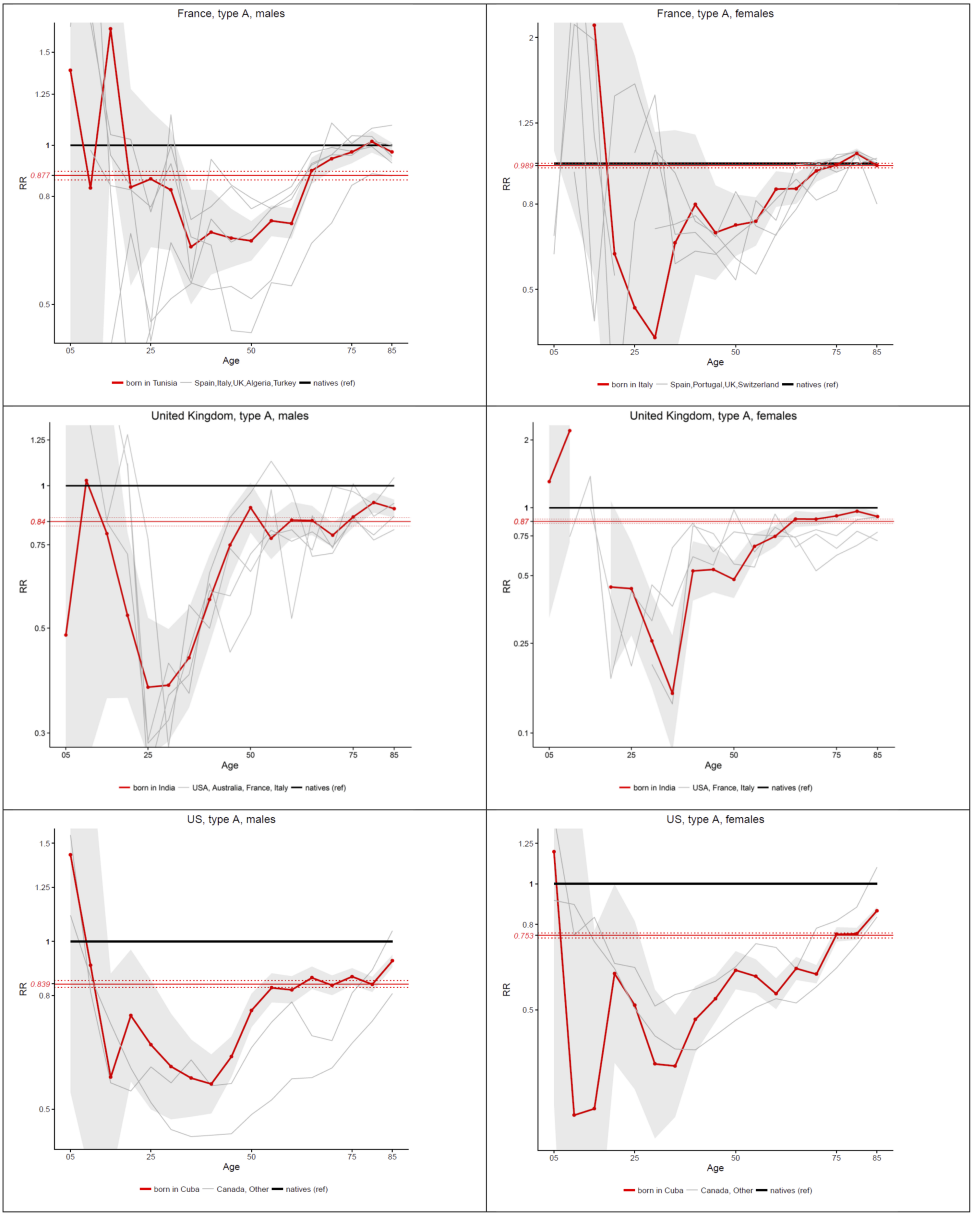
Age-specific and age-standardized mortality ratios (Foreign-born vs Native-Born) in France (2005–09), the UK (2010–11) and the US (2008–10), by sex, for countries of origin with general U-shape pattern.

This overall age pattern hides a great amount of heterogeneity by country of origin, as indicated by the gray lines in the background for Fig 2. Nonetheless, when focusing on individual countries, important regularities emerge. In Fig 3, we present individual countries with an age pattern of relative migrant mortality that is similar to what is observed for all migrants combined. Large countries of origin are represented in this figure, which is expected given the weight that these countries play in the overall pattern presented in in Fig 2. In France, migrants groups that follow this general pattern are males born in Algeria, Italy, Spain, Tunisia, Turkey and the UK, and females born in Italy, Portugal, Spain, Switzerland and the UK. In the UK, males born in Australia, France, India, Italy and the US, and females born in the same countries except Australia follow this pattern. In the US, all migrant groups for whom we have information except individuals born in Mexico present a general U-shape pattern. A detailed analysis of each country is beyond the scope of this paper, but it is quite remarkable that this age pattern applies to such diverse groups of migrants.

**Fig 4:**
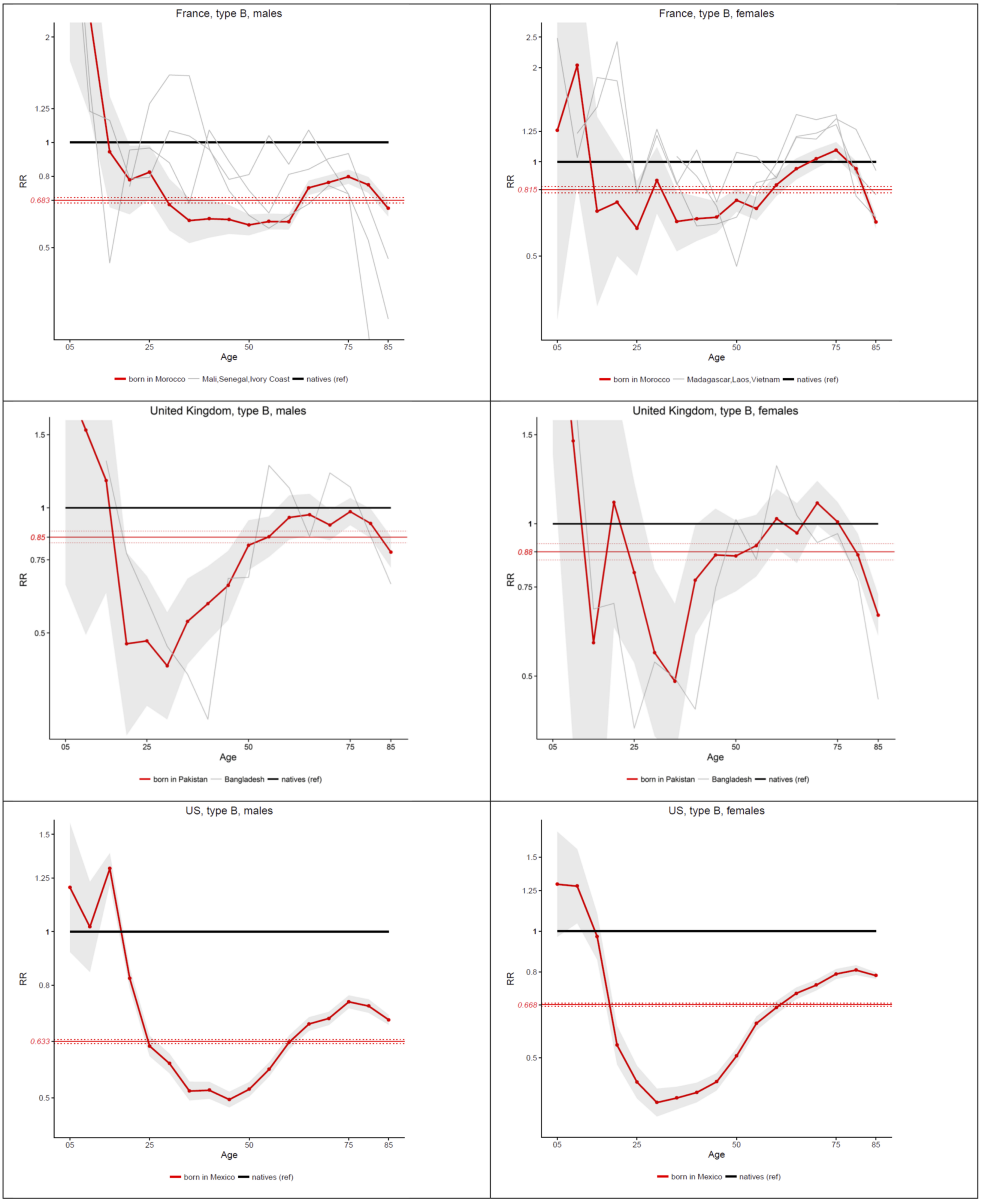
Age-specific and age-standardized mortality ratios (Foreign-born vs Native-Born) in France (2005–09), the UK (2010–11) and the US (2008–10), by sex, for countries of origin with a decline in relative mortality at older ages.

Fig 4 shows a number of individual migrant populations for whom the pattern of relative mortality deviates substantially from the general pattern presented in Figs 2 and 3. Specifically, these migrant populations experience a decline in their risk ratio at older ages, starting around age 75. In France, we find such patterns among males born in Morocco, Senegal, Mali and Ivory Coast, and among females born in Morocco, Madagascar, Laos and Vietnam. In the UK, this pattern appears clearly among males and females born in Bangladesh and Pakistan. In the US, this pattern is visible among Mexican-born males, and, to a lesser extent, among Mexican-born females.

Note that there are also a number of migrant groups in France and the UK not shown in Figs 3 and 4 who exhibit a rather large amount of random variation around one in their mortality ratio due to their small population size, and for whom the specific shape of the age pattern is thus not well defined. It is interesting, nonetheless, that when merging migrant populations by region of origin, an overall U-shape pattern quickly emerges (results not shown). Migrants from Eastern Europe, however, are one exception; they present excess mortality throughout the life course, especially at adult ages. These unusual groups obviously do not follow the general patterns shown in Figs 3 and 4.

## Discussion

This paper demonstrates that far from being constant over age, relative migrant mortality presents large age variations that are often ignored in the migrant mortality literature. This age variation presents some striking similarities across heterogeneous migrant groups and host countries. The age pattern that is most systematic is a U-shape pattern, with a minimum reached among migrants aged 45. This systematicity suggests that similar, general mechanisms operate to explain the relative mortality of migrants across a variety of contexts.

Among the various explanations discussed earlier in the paper, the explanation most consistent with the observed patterns is in-migration selection effects. Indeed, the steep initial decline with age in the risk ratio (often starting from a situation of excess mortality) corresponds to a transition from children who most often arrive as dependents and may not be subject to strong selection forces, to young adults who arrive in large numbers starting at age 20 and profoundly modify the composition of the foreign-born population. As such, this decline reflects a compositional change of the migrant population rather than genuine age effects. The increase with age in the risk ratio after age 45 is consistent with a “wearing off” of the initial selection effects as mean duration of residence in the host country increases, unmitigated by new arrivals which become negligible after age 45.

This U-shape pattern could also be explained by “cultural effects” (which, as discussed earlier, emphasizes the role of health behaviors and social networks), given that this mechanism is expected to produce a similar U-shape pattern. Without additional information, it is difficult to determine which of these two explanations is most relevant. However, it is remarkable that the U-shape pattern is prevalent among migrant groups as diverse as Canadian-born vs. Mexican-born migrants in the US, or Tunisian-born vs. UK-born individuals in France, i.e., immigrant groups that are likely to differ from one another both in terms of prevailing norms regarding health behaviors and integration in immigrant communities in host countries. If cultural effects were the dominant explanation for the migrant mortality advantage, we would expect less pronounced U-shape patterns among migrant groups that are more similar with the host population in terms of cultural norms (e.g. Canadian-born migrants in the US) than among migrant groups that are less similar (e.g., Mexican-born migrants in the US). The pervasiveness of the U-shape pattern among diverse migrant groups suggests that the in-migration selection effects may be a more powerful force than “cultural effects” for explaining the patterns presented in this paper.

For countries experiencing a decline in the risk ratio at older ages, it is difficult to tell if this pattern is explained by out-migration selection effects or data artifacts (such as the exclusion of resident deaths occurring abroad, and age misreporting). Nonetheless, the timing of these declines (starting around age 75), their steepness, and the fact that they disproportionately affect migrants born in less-developed countries is perhaps more suggestive of data artifacts than selection effects. Indeed, there tends to be little international out-migration past age 75, so differences in health status between movers and stayers would have to be extremely large to produce such declines. More likely, these declines are due to a combination of age misreporting and the exclusion of deaths occurring abroad among individuals counted as habitual residents of the host country.

Overall, the out-migration selection effects (“salmon bias”) explanation is poorly supported by the patterns presented in this paper. For most countries, the risk ratio increases with age after age 45 or so, which is not consistent with what we would expect if the salmon bias was a dominant mechanism. Unhealthy remigration, if occurring, seems to be dwarfed by other processes such as wearing off of initial in-migration selection effects or negative acculturation.

One limitation of this study is that while the mechanisms we observe operate over the life course, age profiles of relative migrant mortality are examined in a cross-section. It is possible that earlier cohorts of migrants faced different conditions, explaining their higher relative mortality today, as they reach old ages, than later cohorts of migrants whom we observe at younger ages today. Nonetheless, the pervasiveness of the U-shape pattern across different migrant groups in different host countries suggests that cohort effects are not playing a dominant role. Cohort effects, if important, would be expected to present more variability by migrant group and host country.

It is important to keep in mind that large mortality differentials, when measured in relative terms (as we do here), are rarer at ages where mortality is high than at ages where mortality is low [61]. This makes the decreases in relative mortality prior to age 45 all the more significant, because these decreases occur at ages where mortality rates are increasing with age. This also makes the declines in risk ratios at older ages (Fig 4) particularly significant. For these migrant groups, the mortality advantage increases both in relative and in absolute terms. As for migrant groups showing patterns of mortality convergence at older ages, it is likely that part of the convergence is explained by this “level” effect.

Overall, this paper demonstrates the importance of documenting age variations in the relative mortality of migrants. Examining age-standardized or age-adjusted measures hides the scale of the advantage, which at mid-adult ages appears to be much larger than typically documented in the literature. It also hides a rather common pattern of excess mortality at younger ages, which is not apparent when all ages are combined. Finally, examining age patterns helps advance our understanding of the underlying explanations for the MMA. While a given explanation may be consistent with an “average” advantage across ages, it may not resist the examination of age patterns. The “salmon bias” hypothesis is a prime example of this. On the other hand, the in-migration selection hypothesis gains support when examining age variations in risk ratios as opposed to age-adjusted risk ratios.

The results of this study also suggest important differences, in terms of risk of death, between migrants who arrived as children vs migrants who arrived as adults. As we show, most migrant groups exhibit excess mortality prior to ages 15–20. This reflects an underlying vulnerability among child migrants (sometimes called the 1.5 generation) which is likely to be retained throughout the life course but is difficult to detect in aggregate mortality rates at adult ages due to compositional changes resulting from the arrival of adult migrants. This has implications for studies seeking to evaluate the impact of duration of stay on mortality. Indeed, migrants who arrived as children, when older, will carry with them long durations of stay. If, as suggested here, they also are more vulnerable, these individuals will likely play a large weight in observed relationships between duration of stay and mortality. For them, however, a lack of positive health selection may be a more important mechanism than duration effects per se. Due to this heterogeneity, individual-level analyses of the impact of duration of stay on mortality outcomes should either exclude migrants who arrived, say, prior to age 15, or treat them separately.

## Conclusion

This paper demonstrates the existence of important regularities in how foreign-born vs. native-born mortality ratios vary by age. Using data from France, the US and the UK for periods around 2010, we find similarities in age patterns of mortality ratios across a diversity of migrant groups and host countries. The most systematic age pattern we find is a U-shape pattern: at the aggregate level, migrants often experience excess mortality at young ages, then exhibit a large advantage at adult ages (with the largest advantage around age 45), and finally experience mortality convergence with natives at older ages. The explanation most consistent with this pattern is the “in-migration selection effects” explanation. By contrast, the “out-migration selection effects” explanation is poorly supported by the observed patterns. Our age disaggregation also shows that migrants at mid-adult ages experience mortality advantages that are often far greater than typically documented in this literature. Overall, these results reinforce the notion that migrants are a highly-selected population exhibiting mortality patterns that poorly reflect their living conditions in host countries.
